# Characterizing diverse maize varieties under organic cultivation: phenotypic, yield, and canopy data from VIT Vellore

**DOI:** 10.1016/j.dib.2024.110367

**Published:** 2024-03-26

**Authors:** Sandhya Prakash, Sujatha R, Venkataramana B, T. Pradeesh Kumar

**Affiliations:** a Research Scholar, Data Science, Vellore Institute of Technology, Vellore, Tamil Nadu, India; b School of Computer Science Engineering and Information Systems (SCORE), Vellore Institute of Technology, Vellore, Tamil Nadu, India; c School of Advanced Sciences (SAS), Vellore Institute of Technology, Vellore, Tamil Nadu, India; d VIT School of Agricultural Innovations and Advanced Learning (VAIAL), Vellore Institute of Technology, Vellore, Tamil Nadu, India

**Keywords:** Maize, Genotypes, Plant traits, Kernel weight, Yield data

## Abstract

Maize is an important food source standing first in production around the world. It has become an essential raw material in most of the food processing industries and is extensively cultivated for its post-harvest by-products. So, it is necessary to develop genotypes adapted to varied climate and soil conditions favoring its productivity. Assessing the varietal performance across fields is essential in crop development. Here, we present a dataset of phenotypical and yield attributes of maize. We cultivated maize organically in an agricultural farm of Vellore Institute of Technology, Vellore during the late kharif season of 2023. A total of eight varieties were selected for cultivation, with four recent gazette varieties from IIMR, Punjab, and four locally cultivated varieties. The crop was sown in randomized block design with four replications and eight varieties taken as treatments. We randomly selected 20 plants from each treatment in a way that 5 plants were chosen from each replication and tagged them. Thus, we obtained 160 individual crop data from the overall maize population. The dataset covers: (1) vegetative traits (2) yield traits (3) canopy temperature (CT) and (4) chlorophyll (Ch) readings. Significant difference between genotypes was determined using ANOVA (FRBD). This dataset can be incorporated in future research focused on the breeding and improvement of chosen varieties and can also be utilized for building deep learning models.

Specifications Table

**Table:** 

Subject	Agronomy, Data Engineering, Applied Machine Learning
Specific subject area	Organic cultivation, varietal variation, maize phenotype, yield parameters
Data format	Raw, Analysed
Type of data	Tables, Figures
How data were acquired	Manual and instrument measurements: • Plant height, plant height at cob, leaf length, leaf breadth, cob length, and cob width were measured using measuring tape. • Number of - leaves, nodes, cobs, and tassels were counted data • Ear weight - with and without sheath was measured using a weighing balance. • Rows per ear and kernels per row were counted manually. • The shelled kernel weight of each ear was weighed using a weighing balance. • Canopy temperature (CT) measured twice per week for five weeks from to using FLIR – E8 thermal imaging camera. • chlorophyll (Ch) readings were measured once a week for five weeks using a V-TECH chlorophyll meter.
Description of data collection	• Tagged plant measurements were taken on the days prior to harvest and the corresponding ears were collected. Ear weight with and without sheath was estimated post-harvest, and then the ears were dried to a moisture content of 12 %. Rows/ear and kernels/row were counted before shelling to estimate the whole kernel weight of each ear. • Canopy temperature of flag leaves was measured from 11.00a.m. – 1.00p.m. i.e., during peak sunshine hours • Chlorophyll measurements were taken during 8.00 – 10.00a.m. i.e., when photosynthesis occurs.
Data source location	Land: Sevur farm Latitude: 12.97′ N Longitude: 79.19′ E Institution: Vellore Institute of Technology City: Vellore, Tamil Nadu Country: India
Data accessibility	Repository name: Mendeley Data Data identification number: 10.17632/6py9v57sf2.1 Direct URL to data: https://data.mendeley.com/datasets/6py9v57sf2/1

## 1 Value of the Data

• This data can assist breeders and industrialists in determining the yield stability and area to be cultivated, as maize requires arable soil for efficient growth, this data can be used to analyze how the specific genotype performs under unsuitable soil types as all the measurable physiological and yield traits were estimated.
• The information may be utilized in a variety of maize management research, including agricultural, germplasm improvement, and environmental investigations. Because, it is essential to test the genotype's growth in varied soil and climatic conditions during varietal trials.
• To enhance decision assistance for smallholder farmers, the dataset may be used to evaluate technologies such as modeling, machine learning, and Big Data mining. The data ensemble could be used in simulation for forecasting future trends in population increase, enhance planning techniques, and mitigate risks imposed by climatic uncertainty. Precision farming and decision support systems can be made accessible by machine learning, wherein this data can be used to train algorithms to find connections between yield results and crop management strategies.
• Once there is a bigger corpus of field trials documenting the impacts of varied soil and climate on these genotypes, the data may be valuable in meta-analyses, by conducting intensive dataset analytics to identify patterns, trends, and insights that influence research priorities, policy decisions, and agricultural innovation, ensuring food security and sustainable maize production.
• The results demonstrated the impact of various phenotypical attributes on kernel weight and yield components, which can be utilized to assess the association between our variable of interest (i.e., Kernel weight, Kernels per ear) and other physiological attributes.

## 2 Background

We focussed on gathering complete data on eight diverse maize varieties, including both phenotypical features and yield data to reveal varietal variations in maize performance by carefully assessing phenotypic features such as plant height, leaf shape, and flowering patterns, as well as quantitative yield measures. This dataset can help researchers gain a better understanding of the genetic and environmental variables that influence maize development and yield. The study is important for agricultural science since it provides vital insights into crop enhancement techniques, sustainable farming practices, and guaranteeing food security in a variety of agricultural situations [[5], [6]].

## 3 Data Description

Crop yield potential varies according to soil and environmental conditions. Maize needs well-drained soil types for better growth. It is essential to conduct varietal trials over various locations to test plant growth and yield stability i.e., the interaction between genotypes and environment, before the transfer of knowledge to farmers for better adoption of improved cultivars. Thus, experimented to compare the varietal performance of new hybrids with locally cultivated ones in our region. The dataset is a single table with four major classes: vegetative traits (8 labels), ear traits (7 labels), canopy temperature (5 labels), and chlorophyll readings (5 labels) measured over the crop period. Each label consists of 160 tagged plant measurements of eight treatments with each treatment being replicated four times [[3], [4]].

## 4 Experimental Design, Materials and Methods

### 4.1 Plant materials

The genotypes cultivated were IMH 222, IMH 223, IMH 224, IMHSB 19 KB – 2, Shivani 1980, Pioneer 30BS307, RMH 3414 and RMH 3591. The first four were purchased from IIMR, Punjab, and the other four varieties were purchased from a local seed center.

**Genotype information:**

(1) **IMH 222:** Parentage – BML 7 x IML 14, Season – Rabi, Maturity – Medium, Zone – NWPZ (North Western Plains Zone), Features – Superior yield (17.53 %), Input responsive, Resistant to Maydis leaf blight and Fusarium stalk rot (FSR); has modest resistance to Charcoal rot, *Chilo partellus* bug, and Turcicum leaf blight.
(2) **IMH 223:** Parentage – BML 7 x IML 15, Season – Rabi, Maturity – Medium, Zone – NWPZ (North Western Plains Zone), Features – Superior yield (20.89 %), Input responsive, Resistant to Maydis leaf blight and Fusarium stalk rot (FSR); has modest resistance to Charcoal rot, *Chilo partellus* bug, and Turcicum leaf blight.
(3) **IMH 224:** Parentage – IML 16 x IML 22, Season – Kharif, Maturity – Medium, Zone – NEPZ (North Eastern Plains Zone), Features – Superior yield (13.49 %), Input responsive, Resistant to Fusarium stalk rot (FSR); has modest resistance to Charcoal rot, *Chilo partellus* bug, Maydis leaf blight and Turcicum leaf blight. (*Cultivars – ICAR-Indian Institute of Maize Research*, n.d.)
(4) **IMHSB 19KB – 2:** Variety – Baby corn, Maturity – Medium, TSS – 7.7${}^{\circ }$ brix, Zones – NHZ (North Hill zone), Peninsular Zone (PZ), NEPZ (North Eastern Plains Zone), NWPZ (North Western Plains Zone) and CWZ (Central Western Zone).
(5) **Shivani KSMH 1980:** Season – Kharif, Maturity - Medium, Features – has purple anthers and silk, non-lodging, drought tolerant, high yield potential and is also referred as Hybrid Cotton.
(6) **Pioneer 30B5307:** Maturity – Medium, Features – has slight sweet taste, Resistant to rust and blight diseases.
(7) **RMH 3414:** Features – Disease tolerant, high yielding, needs less management practices and is suited to all weather conditions.
(8) **RMH 3591:** Maturity - Early to medium, Features – drought tolerant and non-lodging.

### 4.2 Description of experimental location and design

#### 4.2.1 Planting and harvesting procedures

**Fig. 1:**
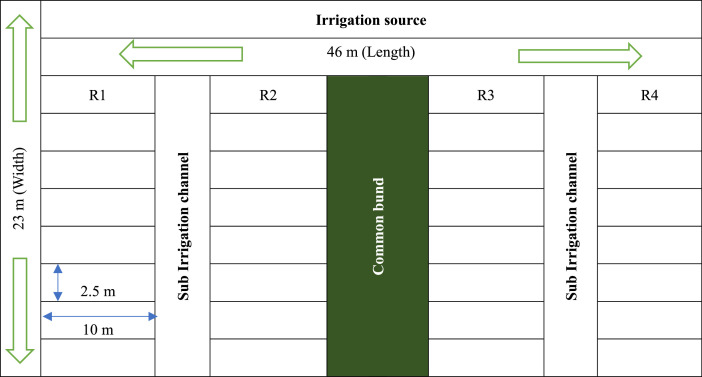
Experimental field layout. [Single Plot Size - 10 × 2.5 *m* = 25 m^2^; Normal Management - 8 Treatments & 4 Replications; Length – 10 (Plot length) + 1.5 (Sub Irrigation Channel) + 10 + 2 (Common Bund) + 10 + 1.5 (Sub Irrigation Channel) + 10 = 45 m; Width – 2.5 (Plot Width) + 2.5 + 2.5 + 2.5 + 2.5 + 2.5 + 2.5 + 2.5 + 2 (Main Irrigation Channel) = 22 m].

**Fig. 2:**
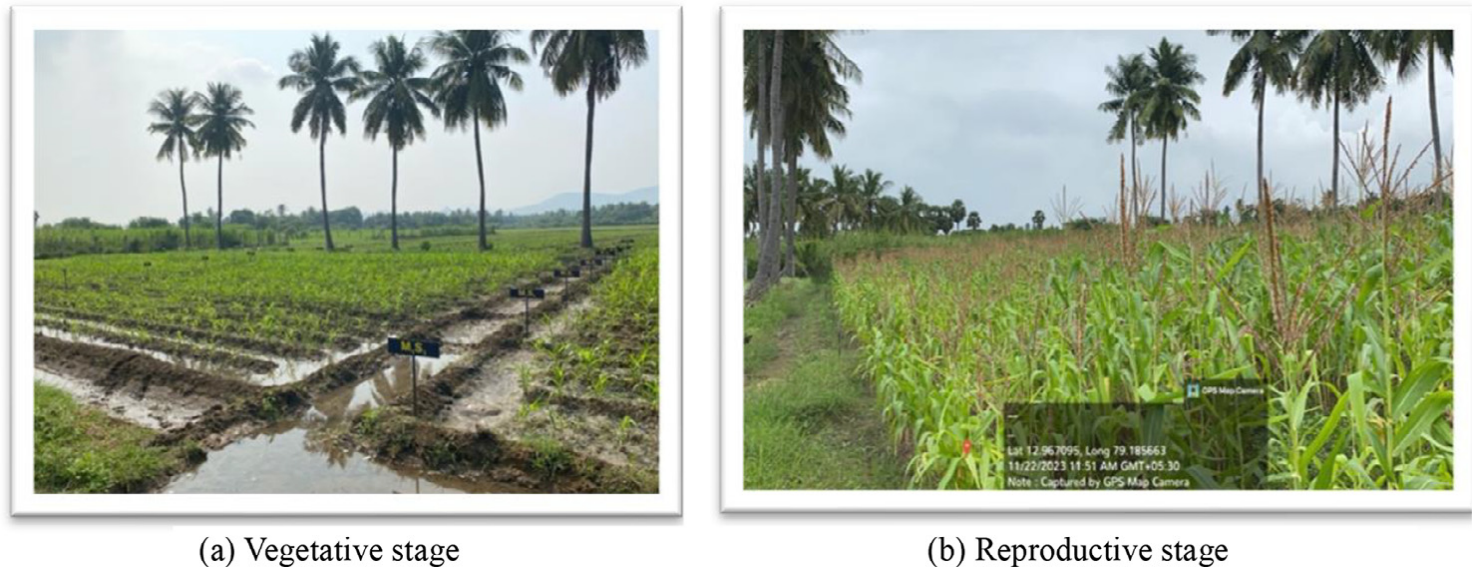
Images of crop growth stages.

The experiment was conducted in the Sevur farm of VIT, Vellore located in the Korandhaangal area at latitude and longitude of 12.97′ N and 9.19′ E respectively. We selected eight genotypes taken as treatments (four new genotypes - T1 to T4 and four locally cultivated one - T5 to T8). The seeds were sown in ridges and furrows formed in layout of Randomized Block Design (RBD) with four replications each. The whole field size was 26 cents with each plot size of 25m^2^ (2.5 m Length x 10 m Width). The planting was carried out in the late kharif season of September 2023 with plant spacing of 60 × 25 cm. The experimental layout is presented in Figs. 1 and 2.

As we adopted RBD we chose a number of five plants at random from each replication (5 * 4) which accounted to 20 plants in total for each replication and 160 (20 * 8) plants in whole field as representative of the whole experimental field. The plots were labelled with their corresponding genotype and replication e.g., T1R1 to T8R4. The selected plants were tagged at 5 – leaf stage using plant labels indicating their number (T1R1 – 1) from 1 to 20 for easier identification. Measurements were taken only on the tagged plants.

**Fig. 3:**
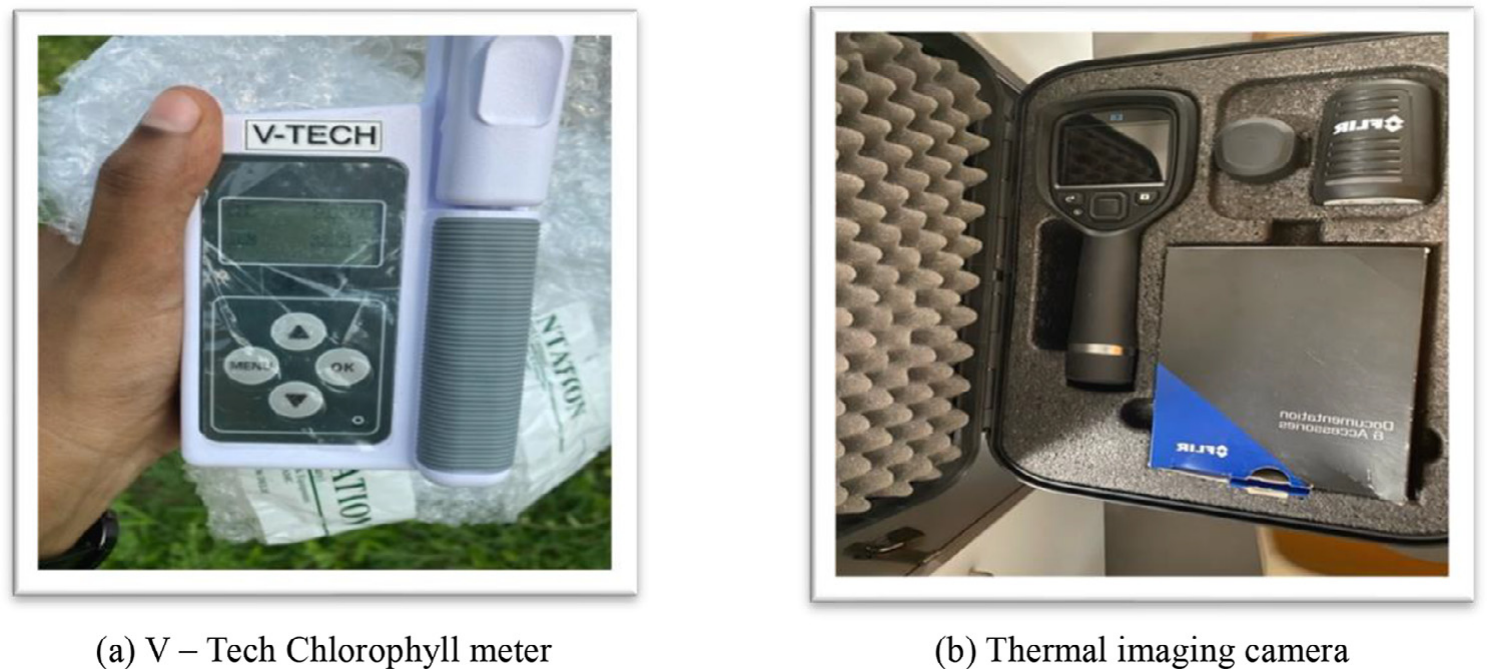
Instruments used for data collection.

Chlorophyll measurements were taken once in a week from tassel initiation till harvest during 7.00 – 9.00am i.e., during efficient photosynthesis hours for a period of five weeks. Canopy temperature was measured using thermal imaging camera twice in a week for the same period as chlorophyll readings and the values were averaged to a week during 11.00am to 1.00pm i.e., during peak sunshine hours. The images of Chlorophyll meter and Infrared Thermometer used for measurements is presented in Fig. 3. The ears of tagged plants were covered with butter paper cover around blister stage to avoid ear destruction by parrots. All plants except tagged ones were harvested and cut-down first such that it was easier to measure the vegetative traits of tagged plants. After measuring the attributes, the ears were harvested and kept in the same butter paper cover along with the tags. The ears were then dried to a moisture content of 12 % prior estimating ear attributes.

#### 4.2.2 Soil and climatic conditions

Soil type of our field is clay-loam (with major composition of clay) which is mostly not suitable for maize cultivation especially during rainy seasons which posed a major hinderance during our research. Water logging became a major problem during crop growth. The spacing adopted was 60 × 25 cm. Life irrigation was carried post sowing on mid-September. The field was irrigated twice every week for 45 days followed by once per week to none when rain started. We top dressed the soil two weeks before harvest as soil eroded during rain because of cyclone.

### 4.3 Data collection

During crop growth canopy temperature and chlorophyll measurements of tagged plants were measured from the tassel initiation stage till harvest. Prior harvest plant attributes were measured and recorded. The ears of separate genotypes were harvested on different dates upon ear maturity, marked, and dried to measure ear attributes. We selected kernel weight (g) for yield comparison between genotypes and classified them under three labels with KW of 0–10 as poor yielding, 10–30 as good yielding, and 30–60 as best yielding genotypes and plants with plant height of 20–40 as poor growth, 40–60 as moderate growth, 60–90 as optimal grown plants. The dataset variables collected and their descriptive statistics have been presented in Tables 1 and 2 respectively.

**Table 1:** Variables in the dataset with their description and units.

Traits	Unit of measurement
(1) Vegetative traits	
Plant number (PN)	Count data
Plant height (PH)	mm
Number of leaves (NOL)	Count data
Number of nodes (NON)	Count data
Number of cobs (NOC)	Count data
Number of tassels (NOT)	Count data
Plant height at cob (PHC)	mm
Leaf length (LL)	mm
Leaf breadth (LB)	mm
(2) Yield traits	
Ear weight with sheath (CWS)	gms
Ear weight without sheath (CWOS)	gms
Ear length (EL)	mm
Ear width (EW)	mm
Rows per ear (RE)	Count data
kernels per row (KR)	Count data
kernel weight (KW)	gms
(3) Canopy temperature (CT)	°C
(4) Chlorophyll (Ch) readings	CCI (Chlorophyll Content Index) units
(5) Treatment	Genotypes

**Table 2:** Descriptive statistics of variables.

	Min.	Max.	Mean	Median	Mode	1st Qu.	3rd Qu.	SD
PH	41.50	89.50	66.90	67.50	63,71,78	60.88	73.05	8.75
NOL	10.00	15.00	11.97	12.00	11.00	11.00	13.00	1.14
NON	10.00	15.00	12.27	12.00	13.00	11.00	13.00	1.20
NOC	1.00	2.00	1.00	1.00	1.00	1.00	1.00	0.08
NOT	3.00	17.00	9.55	9.00	9.00	7.00	12.00	3.36
PHC	11.00	35.00	21.43	21.00	21.00	17.57	24.70	4.64
LL	17.50	33.20	25.61	25.80	27.00	23.15	27.70	3.51
LB	1.90	4.50	2.95	2.90	2.90	2.68	3.20	0.44
EWS	13.00	199.00	69.12	64.00	60.00	49.00	82.00	30.79
EWOS	5.00	126.00	51.87	47.50	30,40,45,55,60	37.00	64.00	23.04
EL	1.90	6.50	4.14	4.20	3.80	3.50	4.80	0.93
EW	0.30	54.00	4.42	4.20	4.50	3.68	4.60	4.02
RE	0.00	36.00	20.83	22.00	24.00	16.75	24.25	6.52
KR	0.00	16.00	12.71	14.00	14.00	12.00	14.00	2.63
KW	0.50	57.00	16.26	15.50	8,11,21	7.75	23.00	11.38

Min. - Minimum value; Max. – Maximum value; SD - Standard Deviation; 1st Qu. – first quartile; 3rd Qu. – third quartile.

**Data collection instruments and calibration:**

Chlorophyll meter needs to be verified first prior checking. The instrument has to be placed on some random leaf for calibration, then it is placed on the flag leaf of tagged plants for measurement.

FLIR thermal imaging camera with a resolution of 76,800 pixels was used to measure canopy temperature. The instrument needs a full-five minute for loading as it stores thermal images of leaves along with temperature. After initiation of instrument, it can be placed at a viewing angle of 45${}^{\circ }$ near the flag leaf for measurement. It stores the images of all leaves it measures which can be accessed anytime which can be shared and saved.

(1) Plant height (PH – from tassel tip to plant base), Plant height at cob (PHC – plant base to node at which ear is formed), Leaf length (LL – flag leaf), Leaf breadth (LB – flag leaf), Ear length (EL – dehusked ears tip to base) and Ear width (EW - dehusked ears) – were measured using measured using measuring tape.
(2) Number of leaves (NOL), Number of nodes (NON), Number of cobs (NOC), Number of tassels (NOT), Rows per ear (RE) and Kernel per row (KR) – were counted manually.
(3) Ear weight with sheath (EWS), Ear weight without sheath (EWOS) and Kernel weight (KW – shelled kernels) – were measured using weighing scale (SF – 400).

### 4.4 Statistical analysis

The collected data was analyzed using R software. The ANOVA table of FRBD shows the replication vector, fact.A (genotypes), fact.B (attributes) and fact.A*fact.B (genotypes*attributes) were statistically significant. The within-treatment difference might be because certain tagged plants were near the border and were subjected to heavy water stagnation which affected the crop growth. Factor variation was estimated using the Least Significant Difference (LSD) test at a 5 % significance level. The R square of 0.951 indicates that the model explains 95.1 % of the variation present in the dependent variable. RMH 3414 (T7) performed significantly better than other genotypes in all attributes and significantly higher ear weight. The results of FRBD is presented in Table 3. Canopy temperature and chlorophyll readings of plants were collected from the tassel initiation stage to the harvest stage and the readings collected during ear initiation had a positive association with kernel weight [[1], [2]].

**Table 3:** Factorial Randomized Block Design (FRBD) - ANOVA.

	Df	SS	MS	F value	Pr(>*F*)
Replication vector	18	25,397	1410.9	39.699	< 2.20E-16 ***
fact.A (Treatment)	6	3468	577.9	16.2613	< 2.20E-16 ***
fact.B (Attributes)	14	201,989	14,427.8	405.9519	< 2.20E-16 ***
fact.A:fact.B	84	13,224	157.4	4.4296	< 2.20E-16 ***
Residuals	357	12,688	35.5		
LSD	2.140		R^2^	0.951	

Df - Degrees of freedom; SS – Sum of Square; MS – Mean Sum of Square; *** *p* < 0; LSD – Least Significant Difference; R^2^ – coefficient of determination; fact.A, fact.B – main effect; fact.A:fact.B – interaction effect.

## Limitations

N/A.

## Ethics statement

N/A.

## CRediT authorship contribution statement

**Sandhya Prakash:** Conceptualization, Data curation, Formal analysis, Writing – original draft. **Sujatha R:** Investigation, Writing – review & editing, Validation. **Venkataramana B:** Funding acquisition, Project administration, Writing – review & editing. **T. Pradeesh Kumar:** Conceptualization, Resources, Supervision.

## Data Availability

Mendeley DataAnalyzing Various Maize Varieties Grown Organically: VIT Vellore's Phenotypic, Yield, and Canopy Data (Original data).
